# Neurosyphilis presenting as autoimmune limbic encephalitis: A case report and literature review

**DOI:** 10.1097/MD.0000000000030062

**Published:** 2022-08-19

**Authors:** Tomotaka Mizoguchi, Makoto Hara, Hideto Nakajima

**Affiliations:** a Division of Neurology, Department of Internal Medicine, Nihon University School of Medicine, Tokyo, Japan.

**Keywords:** autoimmune, diagnostic criteria, limbic encephalitis, neurosyphilis, syndrome

## Abstract

**Rationale:**

Neurosyphilis presenting as limbic encephalitis (LE) is an important differential diagnosis of autoimmune LE (ALE) defined by Graus in 2016. However, data on the clinical differences and similarities between neurosyphilis presenting as LE and ALE are limited. Herein, we report neurosyphilis presenting as ALE that fulfilled the main items of the Graus ALE criteria. Moreover, a literature review of neurosyphilis presenting as LE was performed.

**Patient concerns:**

A 66-year-old Japanese man developed nonconvulsive status epilepticus. He presented with progressive personality change and working memory deficits within 3 months prior to admission. A hyperintense lesion localized in the bilateral medial temporal area was observed on T2-weighted fluid-attenuated inversion recovery brain magnetic resonance imaging. Cerebrospinal fluid analysis showed mild pleocytosis and the presence of oligoclonal band. However, in-house assays did not detect antineuronal antibodies. Electroencephalogram showed lateralized rhythmic delta activity in the right temporal area. The serum and cerebrospinal fluid serological and antigen tests for syphilis had positive results.

**Diagnosis:**

ALE was initially suspected based on the patient’s symptoms and ancillary test findings that fulfilled the Graus ALE criteria. However, based on the positive confirmatory test results for syphilis, a diagnosis of neurosyphilis was eventually made.

**Intervention:**

The patient received intravenous midazolam, oral levetiracetam, and lacosamide to control nonconvulsive status epilepticus. In addition, he was treated with intravenous benzylpenicillin at a dose of 24 million units/day for 14 days.

**Outcomes:**

The patient’s cognitive function relatively improved after antibiotic treatment. However, he presented with persistent mild working memory deficit, which was evaluated with the Wechsler Adult Intelligence Scale, 3rd edition. Therefore, on day 103 of hospitalization, he was transferred to another hospital for rehabilitation and long-term care due to limitations in performing activities of daily living.

**Lessons:**

The present case was diagnosed with neurosyphilis presenting as ALE, but meanwhile, in most case, neurosyphilis presenting as LE developed at a slower progressive rate, and it had a broader or restricted involvement on brain MRI than ALE based on the literature review. Therefore, an appropriate differential diagnosis of LE can be obtained by identifying clinical differences between the 2 conditions.

## 1. Introduction

*Treponema pallidum* infection in the central nervous system (CNS), which is referred to as neurosyphilis, commonly develops in people who have untreated syphilis for several months to years,^[1]^ and can cause chronic-onset cognitive impairment and psychiatric symptoms due to meningovascular syphilis^[2]^ and general paresis.^[2]^ However, in some patients, neurosyphilis can present as limbic encephalitis (LE),^[3,4]^ and this condition is an important differential diagnosis of autoimmune LE (ALE)^[5]^ defined by Graus in 2016 (Graus ALE criteria).^[6]^ Neurosyphilis presenting as LE and ALE can have similar symptoms and clinical features, which include progressive working memory deficits, new-onset seizures, hyperintensities on mesiotemporal T2-weighted fluid-attenuated inversion recovery (T2-FLAIR) magnetic resonance imaging (MRI), and intermittent temporal slow activity on electroencephalogram (EEG).^[6,7]^ However, the similarities and differences between neurosyphilis presenting as LE and ALE are not fully elucidated. Hence, early distinction and initiation of proper treatments are challenging.

Herein, we present a rare case of neurosyphilis presenting as ALE that met the main items of the Graus ALE criteria. Then, a literature review of other cases of neurosyphilis presenting as LE was performed in order to identify and compare the clinical features of neurosyphilis and ALE.

## 2. Case report

A 66-year-old Japanese man with a nonconvulsive status epilepticus (NCSE) was admitted to the hospital. He developed personality change and working memory deficits within 3 months prior to hospitalization. He had a medical history of hypertension but was free of medication. His history was otherwise unremarkable. Upon admission, neurological examination was performed, and results revealed decreased level of consciousness (Glasgow Coma Scale score of 8 [E4V1M3]), left conjugate eye deviation, and reduced voluntary movement on the left side. However, he did not present with fever, syphilis rash, and swelling of systemic lymph nodes.

**Figure 1.**
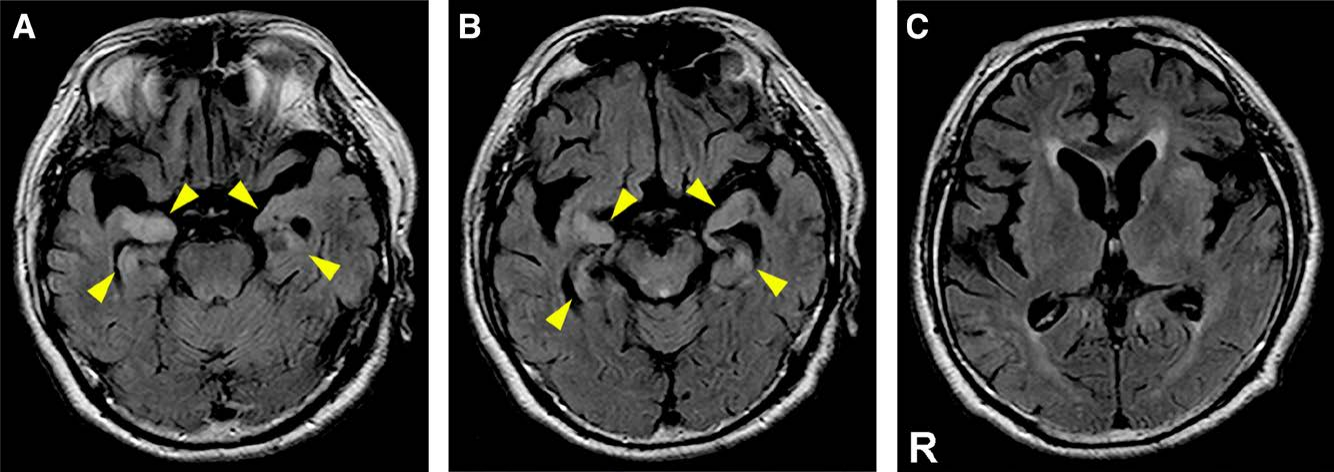
Axial section of T2-FLAIR brain MRI upon admission. (A, B) T2-FLAIR brain MRI upon admission revealed localized high-intensity lesions in the bilateral (highlighted in the right side) medial temporal lobes (indicated by yellow arrows heads). (C) Abnormal signals were not observed in other limbic area such as the insula. MRI = magnetic resonance imaging, T2-FLAIR = T2-weighted fluid-attenuated inversion recovery.

**Figure 2.**
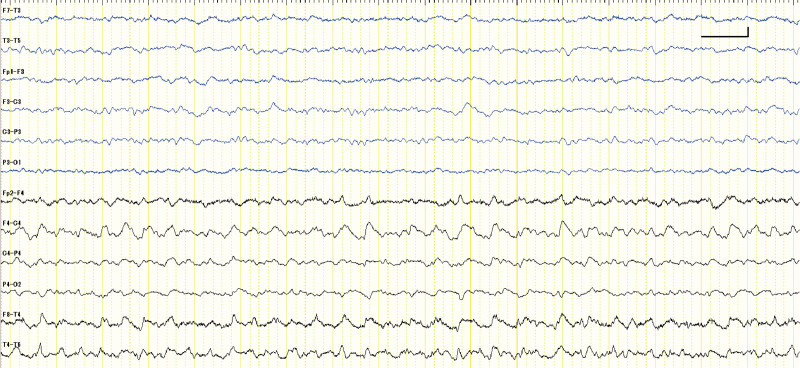
EEG findings upon admission. The initial EEG recording with longitudinal bipolar deviation showed a lateralized rhythmic delta activity in the right temporal area without specific epileptic discharges. EEG montage was recorded with the standard 10–20 electrodes. The blue and black lines indicate recording in the left and right sides, respectively. The vertical and horizontal bars indicate 50 µV and 1 s, respectively. EEG = electroencephalogram.

T2-FLAIR brain MRI revealed high-intensity lesions localized in the bilateral (but highlighted on the right side) mesial temporal lobes that included the hippocampus and amygdala (Fig. 1). EEG revealed lateralized rhythmic delta activity on the right temporal region without specific epileptic discharges (Fig. 2). Blood tests showed a slight increase in white blood cell count (11,000/mm^3^) and C-reactive protein level (0.11 mg/dL). In addition, cerebrospinal fluid (CSF) analysis showed mild inflammation of the CNS as evidenced by high white blood cell counts (25/μL, monocyte predominance) and total protein levels (86 mg/dL). Further, the patient tested positive for oligoclonal band (OCB). Based on highly sensitive polymerase chain reactions, the patient tested negative for both herpes simplex virus and varicella zoster virus DNA in the CSF. The syphilis test revealed positivity for rapid plasma regain (>4 R.U.) and *T pallidum* hemagglutination (150 T.U.) in the serum. In addition, rapid plasma reagin (>4 R.U.), *T pallidum* hemagglutination (2159 T.U.), and fluorescent treponemal antibody were detected in the CSF. The serological tests for human immunodeficiency virus, hepatitis B virus, and hepatitis C virus and T-cell interferon-γ release assay, which is used to detect tuberculosis, had negative results.

We did not detect neuronal intracellular antibodies (Amphiphysin, Hu, Yo, CV2, Ri, Ma2/Ta, recoverin, Tr, GAD65, SOX1, titin, and zic4) related to CNS diseases via an evaluation using line blots (EUROLINE, Euroimmun, Lübeck, Germany). Further, we performed in-house assays that included indirect immunolabeling with rat frozen brain sections and live primary hippocampal neurons.^[8–10]^ Results revealed the absence of antineuronal autoantibodies in the CSF. Moreover, the patient did not present with serum autoantibodies for systemic autoimmune diseases, including antinuclear antibodies, anti-dsDNA, antiphospholipid antibodies, anti-Sjögren syndrome antigen A, anti-Sjögren syndrome antigen B antibodies antibodies, and anti-neutrophil cytoplasmic antibody.

ALE was initially considered because the patient’s symptoms and ancillary test results, particularly brain MRI and CSF analysis, met the main items of the Graus ALE criteria.^[6]^ To obtain an accurate differential diagnosis, serological and antigen tests for syphilis were performed, and the results were positive. Hence, the patient was eventually diagnosed with neurosyphilis masquerading as ALE.

**Figure 3.**
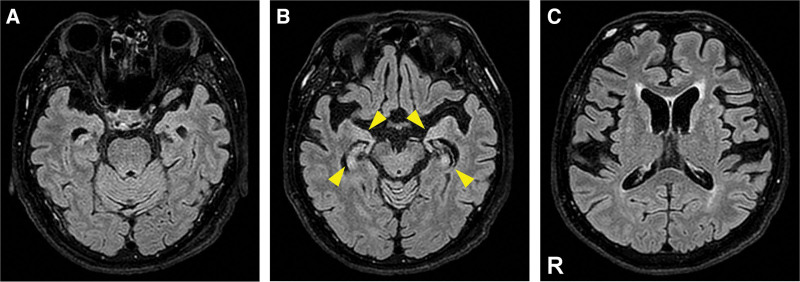
Axial section of T2-FLAIR brain MRI performed on day 75. (A, B) The high-intensity lesions in the bilateral medial temporal lobes on T2-FLAIR brain MRI performed on day 75 partly improved, with weak abnormal signals remaining (indicated by yellow arrows heads). (C) No newly abnormal signals were detected in other limbic area, such as the insula. MRI = magnetic resonance imaging, T2-FLAIR = T2-weighted fluid-attenuated inversion recovery.

To control NSCE, the patient was treated with intravenous midazolam, oral levetiracetam, and lacosamide. He also received intravenous benzylpenicillin at a dose of 24 million units/d for 14 days. His consciousness level gradually improved after treatment. However, mild cognitive dysfunction persisted. His Mini-Mental State Examination score increased from 15 on day 29 to 24 on day 72. In addition, the patient’s cognitive function was evaluated with the Wechsler Adult Intelligence Scale, 3rd edition. He scored full scale IQ 86 (verbal IQ 81: verbal comprehension index 90, working memory index 69; performance IQ 95: perceptual organization index 101, processing speed index 81). This finding indicated the persistence of mild working memory deficits. Regarding the results of follow-up ancillary tests, the value of rapid plasma regain in the CSF was improved (2 R.U.) on day 72. In addition, the high-intensity lesions localized in the bilateral medial temporal lobes on T2-FLAIR brain MRI performed on day 75 partly improved, with weak abnormal signals remaining (Fig. 3). On day 103, he was discharged to another hospital for rehabilitation and long-term care due to limitations in performing activities of daily living. The patient provided written informed consent for publication of this case report.

## 3. Discussion and Literature review

Herein, we report a patient with subacute onset LE who presented with clinical features including the primary symptoms. The ancillary test results fulfilled the Graus ALE criteria.^[6]^ However, the patient was eventually diagnosed with neurosyphilis due to positive CSF serological and specific antigen test results for syphilis. Neurosyphilis is one of the most important differential diagnoses in the Graus ALE criteria.^[6]^ In the current case, the patient met 3 main criteria: subacute progression of working memory deficits (within 3 months) and seizures, high-intensity lesions localized in the bilateral medial temporal lobes on T2-FLAIR MRI, and pleocytosis in the CSF and slow temporal lesion activity on EEG. However, ALE was eventually ruled out due to the identification of alternative causes.

ALE was first described in the 1960s and was found in patients with paraneoplastic causes of LE.^[11]^ Graus et al^[12]^ showed the clinical features of paraneoplastic ALE, which is mainly associated with onconeural antibodies such as Hu and Ma2. The manifestations include subacute onset of seizures (within days or up to 12 weeks), short-term memory loss, confusion, and psychiatric symptoms; neuroradiological evidence of limbic system involvement on MRI, SPECT, or PET; and CSF inflammation, particularly pleocytosis in the CSF. Recently, the number of reports on patients with ALE associated with antibodies against neuronal surface antigens such as leucine-rich glioma-inactivated 1, AMPAR, and gamma-aminobutyric acid receptor type B,^[13]^ some of which were not paraneoplastic, has increased. Therefore, comprehensive screening of antineuronal antibodies was performed, and all results were negative. Based on these findings, Graus et al^[6]^ proposed the Graus ALE criteria in 2016, which include subacute progression of the main syndrome (<3 months), presence of lesions localized in the bilateral medial temporal lobes on T2-weighted FLAIR MRI, and reasonable differential diagnoses including infections (i.e., herpes simplex encephalitis and neurosyphilis), collagen disease, and metabolic disorders.^[6]^ Our patient fulfilled the main inclusion items of the criteria. However, he did not present with any antineuronal antibodies based on in-house and commercial assays.

Neurosyphilis can imitate LE. However, the clinical differences and similarities between neurosyphilis presenting as LE and ALE have not been fully elucidated. Therefore, we performed a literature review of cases of neurosyphilis presenting as LE to validate the characteristics of the disease entity and its differences from ALE.

Cases of neurosyphilis presenting as (or mimicking) LE were searched in PubMed in April 2022. The following terms were used: “neurosyphilis” and “temporal” or “limbic.” In total, 60 cases (50 reports), which include 14 involving general paresis, were matched. Then, the clinical features of the patients were evaluated (Table S1, Supplemental Digital Content, http://links.lww.com/MD/H22, and Supplementary References, Supplemental Digital Content, http://links.lww.com/MD/H23). Result showed that 90% (n = 54) of the patients were men with a median age of 47 (range: 28–73) years. Based on the Graus ALE criteria,^[6]^ 97% (n = 58) of patients presented with symptoms correlated with the limbic system, and 46% (n = 25) developed subacute onset symptoms (≤3 months). However, only 22% (n = 13) of patients had high-intensity lesions in the bilateral medial temporal area on T2-FLAIR MRI, and other patients had unilateral localization or broader involvement in the temporal lobe. In total, 47 (82%) of 57 patients had CSF pleocytosis, and 19 (73%) of 26 patients presented with temporal epileptic or slow-wave activity on EEG. Regarding treatment, 50 patients were managed with antibiotic therapy. Meanwhile, 3 (6%) of 50 patients received combined antibiotics and immunotherapies such as intravenous methylprednisolone and/or intravenous immunoglobulins. Moreover, 22 (55%) of 44 patients experienced complete improvement in their condition, and 20 (45%) of 44 patients only had partial improvement. The number of patients with the disease entity was extremely small. Thus, the effect of immunotherapies could not be evaluated. Table S2 (Supplemental Digital Content, http://links.lww.com/MD/H24) shows the clinical features of the individual cases.

Next, we extracted cases similar to ours in which the patient fulfilled the main Graus ALE criteria.^[6]^ Interestingly, only 3 of 60 patients met the main criteria, and Table 1 shows data about the clinical features of the patients.^[14–16]^ All 3 patients were men with a median age of 50 (35–51) years. Bash et al^[14]^ and Jeong et al^[15]^ reported patients who developed subacute onset working memory deficits, as in our case. The median period of symptom duration was 3 (1–3) months. The ancillary tests revealed CSF pleocytosis. One patient had concomitant human immunodeficiency virus infection.^[16]^ However, our patient did not. All patients were treated with intravenous benzylpenicillin. The condition of 2 patients improved.^[14,15]^ However, the cognitive function of 1 patient did not change.^[16]^ In the present case, NCSE was well controlled with antiepileptic drugs. Nevertheless, mild cognitive dysfunction, as evidenced by working memory deficits, persisted after treatment.

**Table 1:** Cases of neurosyphilis that fulfilled the Graus ALE criteria.

	Authors (year)			
	Bash et al (2001)^[14]^	Jeong et al (2009)^[15]^	Abdelerahman et al (2012)^[16]^	Current case
Age (yr), sex	50, male	35, male	51, male	66, male
Main symptoms	WMD, seizure	WMD, disorientation	Personality change, seizure	Personality change, WMD, NCSE
Symptom duration (mo)	3	1	3	≤3
CSF cell level (/μL)	19	48	220	25
HIV status	N.D	N.D	Positive	Negative
Treatments	Benzylpenicillin	Benzylpenicillin	Benzylpenicillin	Benzylpenicillin
Follow-up period (mo)	4	1	1.5	3.5
Outcome	Improved	Improved	Partly improved	Partly improved (persistence of mild WMD)

ALE = autoimmune limbic encephalitis, CSF = cerebrospinal fluid, HIV = human immunodeficiency virus, NCSE = nonconvulsive status epilepticus, N.D = not described, WMD = working memory deficits.

The mechanisms underlying neurosyphilis presenting as LE should be fully elucidated. OCB in the CSF is often observed in CNS disorders caused by autoimmunity (e.g., multiple sclerosis^[17]^ and AE^[6]^). However, it is also frequently observed in patients with neurosyphilis, similar to our case. A previous study showed that OCB in neurosyphilis represented antibody-synthesis of *T pallidum* in the CSF.^[18]^ Recently, 2 cases of serologically confirmed neurosyphilis presenting as LE accompanied with anti-NMDAR antibodies were published.^[19,20]^ These cases suggest that antibodies might control the symptoms of neurosyphilis. Nevertheless, cases with negativity for NSAs and onconeural antibodies, including the current one, were also reported.^[21]^

However, direct involvement of *T pallidum* in the brain parenchyma is also considered as an alternative mechanism of neurosyphilis presenting as LE.^[5]^ However, no pathologically confirmed cases were reported. Recently, Mao et al^[22]^ reviewed 3 autopsy cases with different subtypes of neurosyphilis, and 1 patient was pathologically diagnosed with meningovascular neurosyphilis and general paresis. Interestingly, Berbel-Garcia et al^[23]^ showed the pathological mechanisms of lesions in the brain parenchyma in general paresis. Further, they showed that high-intensity lesions on T2-weighted imaging might reflect reversible microglial hypertrophy and edema. Cases mimicking viral encephalitis or transient ischemic attacks were more compatible with the preparalytic stage.^[23]^ With consideration of the possible development of general paresis, neurosyphilis presenting as LE might be in the early phase of the disease. However, in our case, this finding could not be confirmed as long-term follow-up brain MRI could not be performed. Thus, further investigations must be conducted to validate if *T pallidum* involvement has regional propensity in the brain parenchyma, particularly in the medial temporal lobes, in patients with neurosyphilis presenting as LE.

In conclusion, we present a rare case of neurosyphilis presenting as ALE. According to the literature review, most cases of neurosyphilis presenting as LE did not fulfill the essential items of the Graus ALE criteria. Namely, only 3 of 60 cases fulfilled the criteria because neurosyphilis was more likely to have a slower clinical progression and broader or more restricted involvement on brain MRI than ALE. Hence, an appropriate differential diagnosis of LE can be obtained based on the clinical differences between the 2 conditions.

## Author contributions

Conceptualization: Tomotaka Mizoguchi, Makoto Hara

Data curation: Tomotaka Mizoguchi, Makoto Hara

Funding acquisition: Makoto Hara

Investigation: Tomotaka Mizoguchi, Makoto Hara, Hideto Nakajima

Supervision: Makoto Hara, Hideto Nakajima

Visualization: Tomotaka Mizoguchi, Makoto Hara

Writing – original draft: Tomotaka Mizoguchi, Makoto Hara

Writing – review & editing: Makoto Hara, Hideto Nakajima

## Supplementary Material



## References

[1] Chow F. Neurosyphilis. Continuum (Minneap Minn). 2021;27:1018–39. 34623102 10.1212/CON.0000000000000982

[2] Marra CM. Neurosyphilis. Continuum (Minneap Minn). 2015;21:1714–28. 26633785 10.1212/CON.0000000000000250

[3] Chow FC Glaser CA Sheriff H. Use of clinical and neuroimaging characteristics to distinguish temporal lobe herpes simplex encephalitis from its mimics. Clin Infect Dis. 2015;60:1377–83. 25637586 10.1093/cid/civ051 PMC4462661

[4] Toudou-Daouda M Filali-Adib A Slassi A. Limbic encephalitis: experience of a Moroccan center. Brain Behav. 2019;9:e01177. 30474361 10.1002/brb3.1177 PMC6346419

[5] Scheid R Voltz R Vetter T. Neurosyphilis and paraneoplastic limbic encephalitis: important differential diagnoses. J Neurol. 2005;252:1129–32. 15789128 10.1007/s00415-005-0812-1

[6] Graus F Titulaer MJ Balu R. A clinical approach to diagnosis of autoimmune encephalitis. Lancet Neurol. 2016;15:391–404. 26906964 10.1016/S1474-4422(15)00401-9 PMC5066574

[7] Serrano-Cardenas KM Sánchez-Rodriguez A Pozueta A. Mesial encephalitis: an uncommon presentation of neurosyphilis: a case report and review of the literature. Neurol Sci. 2018;39:173–6. 10.1007/s10072-017-3109-0 28889343

[8] Hara M Martinez-Hernandez E Ariño H. Clinical and pathogenic significance of IgG, IgA, and IgM antibodies against the NMDA receptor. Neurology. 2018;90:e1386–94. 29549218 10.1212/WNL.0000000000005329 PMC5902781

[9] Hara M Nakajima H Kamei S. Practical approach for the diagnosis of disorders associated with antibodies against neuronal surface proteins. Neurol Clin Neurosci. 2021;9:56–62.

[10] Mizoguchi T Hara M Hirose S. Novel qEEG biomarker to distinguish anti-NMDAR encephalitis from other types of autoimmune encephalitis. Front Immunol. 2022;13:845272. 35242143 10.3389/fimmu.2022.845272 PMC8885512

[11] Corsellis JA Goldberg GJ Norton AR. “Limbic encephalitis” and its association with carcinoma. Brain. 1968;91:481–96. 5723018 10.1093/brain/91.3.481

[12] Graus F Delattre JY Antoine JC. Recommended diagnostic criteria for paraneoplastic neurological syndromes. J Neurol Neurosurg Psychiatry. 2004;75:1135–40. 15258215 10.1136/jnnp.2003.034447 PMC1739186

[13] Asztely F Kumlien E. The diagnosis and treatment of limbic encephalitis. Acta Neurol Scand. 2012;126:365–75. 22713136 10.1111/j.1600-0404.2012.01691.x

[14] Bash S Hathout GM Cohen S. Mesiotemporal T2-weighted hyperintensity: neurosyphilis mimicking herpes encephalitis. AJNR Am J Neuroradiol. 2001;22:314–6. 11156776 PMC7973961

[15] Jeong YM Hwang HY Kim HS. MRI of neurosyphilis presenting as mesiotemporal abnormalities: a case report. Korean J Radiol. 2009;10:310–2. 19412521 10.3348/kjr.2009.10.3.310 PMC2672188

[16] Abdelerahman KT Santamaria DD Rakocevic G. Pearls and oysters: neurosyphilis presenting as mesial temporal encephalitis. Neurology. 2012;79:e206–8. 23233688 10.1212/WNL.0b013e318278b5a1

[17] Polman CH Reingold SC Banwell B. Diagnostic criteria for multiple sclerosis: 2010 revisions to the McDonald criteria. Ann Neurol. 2011;69:292–302. 21387374 10.1002/ana.22366 PMC3084507

[18] Vartdal F Vandvik B Michaelsen TE. Neurosyphilis: intrathecal synthesis of oligoclonal antibodies to Treponema pallidum. Ann Neurol. 1982;11:35–40. 7036846 10.1002/ana.410110107

[19] Qin K Wu W Huang Y. Anti-N-methyl-D-aspartate receptor(NMDAR) antibody encephalitis presents in atypical types and coexists with neuromyelitis optica spectrum disorder or neurosyphilis. BMC Neurol. 2017;17:1. 28056870 10.1186/s12883-016-0787-9 PMC5216582

[20] Beiruti K Abu Awad A Keigler G. Atypical development of neurosyphilis mimicking limbic encephalitis. Int J STD AIDS. 2019;30:194–7. 30253732 10.1177/0956462418797873

[21] Geisler F Smyth M Oechtering J. Auto-antibody-negative limbic-like encephalitis as the first manifestation of neurosyphilis. Clin Neurol Neurosurg. 2013;115:1485–7. 23265560 10.1016/j.clineuro.2012.11.012

[22] Mao C Gao J Jin L. Postmortem histopathologic analysis of neurosyphilis: a report of 3 cases with clinicopathologic correlations. J Neuropathol Exp Neurol. 2018;77:296–301 10.1093/jnen/nly004 29546365

[23] Berbel-Garcia A Porta-Etessam J Martinez-Salio A. Magnetic resonance image-reversible findings in a patient with general paresis. Sex Transm Dis. 2004;31:350–2. 15167644 10.1097/00007435-200406000-00006

