# Supplementary material for: Neurosyphilis presenting as autoimmune limbic encephalitis: A case report and literature review
Source: Medicine (Baltimore). 2022 Aug 19;101(33):e30062. doi: 10.1097/MD.0000000000030062 (PMC9388039; doi:10.1097/MD.0000000000030062)
Supplement: Supplementary file 1 [file medi-101-e30062-s001.pdf]

Supplemental Digital Content 1

# Neurosyphilis presenting as autoimmune limbic encephalitis: A case report and literature review

Tomotaka Mizoguchi, MD

**Supplementary Table 1.** Clinical features of 60 patients, described in the 50 reports, with neurosyphilis presenting as (or mimicking) LE[‡]

| | |
|---|---|
| Median age (range) | 47 (28–73) |
| Male sex (%) | 54 / 60 (90%) |
| Diagnosed with general paresis (%) | 14 / 60 (23%) |
| Symptoms | |
| Related with the limbic system (%) | 58 / 60 (97%) |
| Subacute onset, ≤3 months (%) | 25 / 54 (46%) |
| Abnormal findings on T2-weighted FLAIR MRI | |
| Localized lesions in the bilateral medial temporal area* (%) | 13 / 60 (22%) |
| CSF pleocytosis (%) | 47 / 57 (82%) |
| EEG findings | |
| Temporal epileptic or slow-wave activity (%) | 19 / 26 (73%) |
| Treatments | |
| Antibiotics (%) | 50 / 50 (100%) |
| Immunotherapies (%) | 3 / 50 (6%) |
| Outcomes | |
| Completely improved[#] (%) | 24 / 44 (55%) |
| Partly improved (%) | 20 / 44 (45%) |

[‡] The reviewed 50 literatures are listed in Supplementary References; Supplemental Digital Content 2.

* Unilateral medial temporal lesion and those expanding in other lobes (e.g., frontal, insular, and parietal lobes) were excluded.

[#] This column includes cases with the description "improved."

Abbreviations: CSF: cerebrospinal fluid, EEG: electroencephalogram, FLAIR: fluid-attenuated inversion recovery, LE: limbic encephalitis, MRI: magnetic resonance imaging.
