# Supplementary material for: Neurosyphilis presenting as autoimmune limbic encephalitis: A case report and literature review
Source: Medicine (Baltimore). 2022 Aug 19;101(33):e30062. doi: 10.1097/MD.0000000000030062 (PMC9388039; doi:10.1097/MD.0000000000030062)
Supplement: Supplementary file 2 [file medi-101-e30062-s002.pdf]

Supplemental Digital Content 2

Neurosyphilis presenting as autoimmune limbic encephalitis: A case report and literature review

Tomotaka Mizoguchi, MD

Supplementary References

[1] Angus F, Maysuria H, Bryan CS. Neurosyphilis mimicking herpes simplex encephalitis. *J S C Med Assoc*. 1998;94:315-317.

[2] Denays R, Collier A, Rubinstein M, Atsama P. A 51-year-old woman with disorientation and amnesia. *Lancet*. 1999;354:1786.

[3] Szilak I, Marty F, Helft J, Soeiro R. Neurosyphilis presenting as herpes simplex encephalitis. *Clin Infect Dis*. 2001;32:1108-1109.

[4] Lauria G, Erbetta A, Pareyson D, Sghirlanzoni A. Parenchymatous neurosyphilis. *Neurol Sci*. 2001;22:281-282.

[5] Bash S, Hathout GM, Cohen S. Mesiotemporal T2-weighted hyperintensity: neurosyphilis mimicking herpes encephalitis. *AJNR Am J Neuroradiol*. 2001;22:314-316.

[6] Fujimoto H, Imaizumi T, Nishimura Y, et al. Neurosyphilis showing transient global amnesia-like attacks and magnetic resonance imaging abnormalities mainly in the limbic system. *Intern Med*. 2001;40:439-442.

[7] Silberstein P, Lawrence R, Pryor D, Shnier R. A case of neurosyphilis with a florid Jarisch-Herxheimer reaction. *J Clin Neurosci*. 2002;9:689-690.

[8] Vojvodic NM, Sokic DV, Jankovic SM, Delic S. Isolated episodes of status epilepticus as the manifestation of neurosyphilis: a case report. *Epilepsia*. 2003;44:623.

[9] Marano E, Briganti F, Tortora F, et al. Neurosyphilis with complex partial status epilepticus and mesiotemporal MRI abnormalities mimicking herpes simplex encephalitis. *J Neurol Neurosurg Psychiatry*. 2004;75:833.

[10] Ances BM, Shellhaus R, Brown MJ, Rios OV, Herman ST, French JA. Neurosyphilis and status epilepticus: case report and literature review. Epilepsy Res 2004;59:67-70.

[11] Vieira Santos A, Matias S, Saraiva P, Goulão A. Differential diagnosis of mesiotemporal lesions: case report of neurosyphilis. *Neuroradiology*. 2005;47:664-667.

[12] Scheid R, Voltz R, Vetter T, Sabri O, von Cramon DY. Neurosyphilis and paraneoplastic limbic encephalitis: important differential diagnoses. *J Neurol*. 2005;252:1129-1132.

[13] Li CH, Su CL, Lin WC, Lin RT. Status epilepticus as an initial manifestation of neurosyphilis: a case report. Kaohsiung *J Med Sci*. 2006;22:404-409.

[14] Fadil H, Gonzalez-Toledo E, Kelley BJ, Kelley RE. Neuroimaging findings in neurosyphilis. *J Neuroimaging*. 2006;16:286-289.

[15] Gürses C, Kürtüncü M, Jirsch J, et al. Neurosyphilis presenting with status epilepticus. *Epileptic Disord*. 2007;9:51-56.

[16] Hama K, Ishiguchi H, Tuji T, Miwa H, Kondo T. Neurosyphilis with mesiotemporal magnetic resonance imaging abnormalities. *Intern Med*. 2008;47:1813-1817.

[17] Peng F, Hu X, Zhong X, et al. CT and MR findings in HIV-negative neurosyphilis. *Eur J Radiol*. 2008;66:1-6.

[18] Sesar A, Arias M, Requena I, Pereiro I. Status epilepticus secondary to luetic encephalitis: evolution of neuroimaging findings. *J Neurol*. 2008;255:438-440.

[19] Jeong YM, Hwang HY, Kim HS. MRI of neurosyphilis presenting as mesiotemporal abnormalities: a case report. Korean *J Radiol*. 2009;10:310-312.

[20] Vedes E, Geraldo AF, Rodrigues R, Reimão S, Ribeiro A, Antunes F. Neurosyphilis versus Herpes Encephalitis in a Patient with Confusion, Memory Loss, and T2-Weighted Mesiotemporal Hyperintensity. *Case Rep Infect Dis*. 2012;2012:154863.

[21] Omer TA, Fitzgerald DE, Sheehy N, Doherty CP. Neurosyphilis presenting with unusual hippocampal abnormalities on magnetic resonance imaging and positron emission tomography scans: a case report. *J Med Case Rep*. 2012;6:389.

[22] Saunderson RB, Chan RC. Mesiotemporal changes on magnetic resonance imaging in neurosyphilis. *Intern Med J*. 2012;42:1057-1063.

[23] Abdelerahman KT, Santamaria DD, Rakocevic G. Pearls and oy-sters: neurosyphilis presenting as mesial temporal encephalitis. *Neurology*. 2012;79:e206-208.

[24] Xiang T, Li G, Xiao L, et al. Neuroimaging of six neurosyphilis cases mimicking viral encephalitis. *J Neurol Sci*. 2013;334:164-166.

[25] Derouich I, Messouak O, Belahsen MF. Syphilitic limbic encephalitis revealed by status epilepticus. BMJ Case Rep 2013;2013.

[26] Geisler F, Smyth M, Oechtering J, Tuetuencue S, Klostermann F, Nolte CH. Auto-antibody-negative limbic-like encephalitis as the first manifestation of Neurosyphilis. *Clin Neurol Neurosurg*. 2013;115:1485-1487.

[27] Aizawa H, Yomono H, Kurisaki H. Neurosyphilis presenting as frontal and mesial temporal encephalitis. *Intern Med*. 2013;52:2381-2382.

[28] Mignarri A, Arrigucci U, Coleschi P, Bilenchi R, Federico A, Dotti MT. Temporal lobe abnormalities in neurosyphilis. *Pract Neurol*. 2014;14:449-450.

[29] Takagaki K, Morales MK, Vitantonio D, et al. Periodic Lateralized Epileptiform Discharges (PLEDs) in Patients With Neurosyphilis and HIV Infection. *Clin EEG Neurosci*. 2016;47:247-250.

[30] Bhai S, Biffi A, Bakhadirov K, Prasad S. Mystery Case: A 64-year-old woman with subacute encephalopathy. *Neurology*. 2015;85:e64-65.

[31] Pesaresi I, Sabato M, Doria R, et al. Susceptibility-weighted imaging in parenchymal neurosyphilis: identification of a new MRI finding. *Sex Transm Infect*. 2015;91:489-492.

[32] Tsukita K, Shimotake A, Nakatani M, Takahashi Y, Ikeda A, Takahashi R. [A case of neurosyphilis presenting with limbic encephalitis] (Japanese). *Rinsho Shinkeigaku*. 2017;57:37-40.

[33] Qin K, Wu W, Huang Y, et al. Anti-N-methyl-D-aspartate receptor(NMDAR) antibody encephalitis presents in atypical types and coexists with neuromyelitis optica spectrum disorder or neurosyphilis. *BMC Neurol*. 2017;17:1.

[34] Budhram A, Silverman M, Burneo JG. Neurosyphilis mimicking autoimmune encephalitis in a 52-year-old man. *Cmaj*. 2017;189:E962-e965.

[35] Ikeda S, Yakushiji Y, Eriguchi M, Fujii Y, Ishitsuka K, Hara H. [Neurosyphilis with cerebellar ataxia, personality change and cognitive decline one year after onset of cerebral infarction] (Japanese). *Rinsho Shinkeigaku*. 2018;58:499-504.

[36] Tiwana H, Ahmed A. Neurosyphilis: mighty imitator forays with benign presentation and unique neuroimaging findings. *Sex Health*. 2018;15:358-360.

[37] Jadeja N, Delfiner L, Zarnegar R, Milstein MJ. Infectious Temporal Lobe Encephalitis-Not Just Herpes! *Neurohospitalist*. 2018;8:156-157.

[38] Serrano-Cardenas KM, Sánchez-Rodriguez A, Pozueta A, Pelayo AL, Riancho J. Mesial encephalitis: an uncommon presentation of neurosyphilis: a case report and review of the literature. *Neurol Sci*. 2018;39:173-176.

[39] Skalnaya A, Fominykh V, Ivashchenko R, et al. Neurosyphilis in the modern era: Literature review and case series. *J Clin Neurosci*. 2019;69:67-73.

[40] Toffanin T, Miola A, Follador H, et al. A Case Report of Neurosyphilis Limbic Encephalitis With Reversible Geschwind Syndrome and Mood Disorder. *J Psychiatr Pract*. 2019;25:222-226.

[41] Daey Ouwens IM, Fiolet ATL, Thijs RD, Koehler PJ, Verhoeven WMA. Neurosyphilis Mimicking Autoimmune Encephalitis: A Case Report and Review of the Literature. *Clin Neuropsychiatry*. 2020;17:175-180.

[42] Pisché G, Spitz I, Siffray-Bauer L, Dentel C, Perriard J, Carré S. All that glitters is not gold: A limbic encephalitis due to neurosyphilis. *Rev Neurol (Paris)*. 2021;177:156-157.

[43] Liu C, Zhang Y, Li Y, et al. Neurosyphilis with a rare magnetic resonance imaging pattern confirmed by metagenomic next-generation sequencing: a case report. *Eur J Med Res*. 2022;27:49.

[44] Jum'ah A, Aboul Nour H, Alkhoujah M, Zoghoul S, Eltous L, Miller D. Neurosyphilis in disguise. *Neuroradiology*. 2022;64:433-441.

[45] Berbel-Garcia A, Porta-Etessam J, Martinez-Salio A, et al. Magnetic resonance image-reversible findings in a patient with general paresis. *Sex Transm Dis*. 2004;31:350-352.

[46] Zhang SQ, Wan B, Ma XL, Zheng HM. Worsened MRI findings during the early period of treatment with penicillin in a patient with general paresis. *J Neuroimaging*. 2008;18:360-363.

[47] Yu Y, Wei M, Huang Y, et al. Clinical presentation and imaging of general paresis due to neurosyphilis in patients negative for human immunodeficiency virus. *J Clin Neurosci*. 2010;17:308-310.

[48] Wang X, Yang Y, Wang X, Li C. MRI findings and early diagnosis of general paresis of the insane. *Neurol Res*. 2014;36:137-142.

[49] Ishihara T, Ishihara A, Ozawa T, Sanpei K, Shimohata T, Nishizawa M. [Clinical course and serial brain MRI findings in a patient with Lissauer form of general paresis] (Japanese). *Rinsho Shinkeigaku*. 2015;55:238-242.

[50] Nishina T, Uemori M, Satou T, Asano A. [A case of relapsed neurosyphilis with progressive left hemiparesis] (Japanese). *Rinsho Shinkeigaku*. 2018;58:395-398.

[51] Graus F, Titulaer MJ, Balu R, et al. A clinical approach to diagnosis of autoimmune encephalitis. *Lancet Neurol*. 2016;15(4):391-404.
