# Supplementary material for: Neurosyphilis presenting as autoimmune limbic encephalitis: A case report and literature review
Source: Medicine (Baltimore). 2022 Aug 19;101(33):e30062. doi: 10.1097/MD.0000000000030062 (PMC9388039; doi:10.1097/MD.0000000000030062)
Supplement: Supplementary file 3 [file medi-101-e30062-s003.pdf]

Supplemental Digital Content 3

Neurosyphilis presenting as autoimmune limbic encephalitis: A case report and literature review

Tomotaka Mizoguchi, MD

Supplementary Table 2. Detailed clinical information on the individual cases of neurosyphilis presenting as (or mimicking) LE

Authors[§] (year)	Sex (age)	Main syndrome[#]: Related with limbic system	Main syndrome[#]: Subacute onset (≤ 3 months)	Lesions localized in bilateral medial temporal lobes on FLAIR MRI[#]	CSF pleocytosis[#]	Temporal dominant epileptic or slow-wave activity in EEG[#]	Treatment: Antibiotics	Treatment: Immuno-therapies	Outcomes
Angus F (1998)[1]	M (34)	+	+	-	+	+	+	-	Partly improved
Denays R (1999)[2]	F (51)	+	+	-	+	+	+	-	Improved
Szilak I (2001)[3]	M (55)	+	+	-	+	+	+	-	Partly improved
Lauria G (2001)[4]	M (62)	+	-	-	-	N.D	+	-	Partly improved

Bash S (2001)[5]	M (50)	+	+	+	+	+	+	-	Improved
Fujimoto H (2001)[6]	M (41)	+	-	-	+	-	+	-	Partly improved
Silberstein P (2002)[7]	M (37)	+	-	-	+	N.D	+	-	Partly improved
Vojvodic NM (2003)[8]	M (45)	+	-	+	+	+	+	-	Improved
Marano E (2004)[9]	M (48)	+	+	-	+	N.D	+	-	N.D
Ances BM (2004)[10]	M (41)	+	+	-	+	+	+	-	Partly improved
Vieira Santos A (2005)[11]	M (73)	+	+	-	+	+	+	-	N.D
Scheid R (2005)[12]	M (34)	+	+	-	-	-	+	-	Partly improved
Li CH (2006)[13]	M (41)	+	+	-	+	-	+	-	Partly improved
Fadil H (2006)[14]	M (41)	+	-	-	+	+	+	-	N.D
Gürses C (2007)[15]	M (42)	+	-	+	+	+	+	-	Partly improved

Hama K (2008)[16]	M (51)	+	-	-	+	N.D	+	-	Partly improved
Peng F (2008)[17]	M (42)	+	N.D	-	N.D	N.D	+	-	N.D
Sesar A (2008)[18]	M (57)	+	-	-	+	+	+	-	Partly improved
Jeong YM (2009)[19]	M (35)	+	+	+	+	N.D	+	-	Improved
Vedes E (2012)[20]	M (43)	+	+	-	+	N.D	+	-	Partly improved
Omer TA (2012)[21]	M (55)	+	-	-	+	-	+	-	Improved
Saunderson RB (2012)[22]	M (53)	+	-	-	N.D	N.D	+	-	N.D
Abdelerahman KT (2012)[23]	M (51)	+	+	+	+	+	+	-	Partly improved
Xiang T (2013)[24]	M (43)	+	+	-	+	N.D	+	-	Partly improved
Xiang T (2013)[24]	M (30)	+	+	-	+	N.D	+	-	Partly improved
Xiang T (2013)[24]	M (45)	-	+	-	+	N.D	+	-	Partly improved

Derouich I (2013)[25]	M (50)	+	+	-	+	+	+	-	Improved
Geisler F (2013)[26]	M (66)	+	+	-	+	N.D	+	-	Improved
Aizawa H (2013)[27]	F (46)	+	-	-	N.D	N.D	+	-	Improved
Mignarri A (2014)[28]	F (28)	+	-	+	-	N.D	+	-	Improved
Takagaki K (2014)[29]	M (52)	+	+	-	+	+	+	-	Improved
Bhai S (2015)[30]	F (64)	+	-	-	+	N.D	+	+	Partly improved
Pesaresi I (2015)[31]	M (42)	+	-	+	+	N.D	+	-	Improved
Tsukita K (2016)[32]	M (46)	+	-	+	+	N.D	+	-	Improved
Qin K (2017)[33]	M (39)	+	N.D	-	-	N.D	+	+	Partly improved
Budhram A (2017)[34]	M (52)	+	-	-	+	+	+	+	Improved
Ikeda S (2018)[35]	M (51)	+	-	-	+	N.D	+	-	Improved

Tiwana H (2018)[36]	M (52)	-	-	+	+	N.D	+	-	Improved
Jadeja N (2018)[37]	M (49)	+	+	-	+	+	+	-	N.D
Serrano-Cardenas KM (2018)[38]	M (62)	+	N.D	+	+	N.D	+	-	Improved
Skalnaya A (2019)[39]	M (47)	+	+	-	+	N.D	+	-	Improved
Toffanin T (2019)[40]	F (34)	+	-	-	+	+	+	-	Improved
Daey Ouwen IM (2020)[41]	M (62)	+	-	-	-	+	+	-	Improved
Pisché G (2021)[42]	M (65)	+	N.D	-	+	+	+	-	Improved
Liu C (2022)[43]	M (29)	+	+	-	+	-	+	-	Improved
Jum'ah A (2022)[44]	F (69)	+	+	-	+	N.D	+	-	Improved
Below are the cases who were classified into the subtype of general paresis									
Berbel-Garcia A (2004)[45]	M (47)	+	-	-	-	N.D	+	-	Partly improved

Zhang SQ (2008)[46]	M (52)	+	-	-	+	N.D	+	-	Improved
Yu Y (2010)[47]	M (40)	+	N.D	-	+	+	N.D	N.D	N.D
Yu Y (2010)[47]	M (35)	+	N.D	+	+	-	N.D	N.D	N.D
Wang X (2014)[48]	M (45)	+	-	-	+	N.D	N.D	N.D	N.D
Wang X (2014)[48]	M (37)	+	+	-	+	N.D	N.D	N.D	N.D
Wang X (2014)[48]	M (49)	+	-	-	-	N.D	N.D	N.D	N.D
Wang X (2014)[48]	M (60)	+	-	-	+	N.D	N.D	N.D	N.D
Wang X (2014)[48]	M (46)	+	-	-	+	N.D	N.D	N.D	N.D
Wang X (2014)[48]	M (33)	+	-	-	-	N.D	N.D	N.D	N.D
Wang X (2014)[48]	M (38)	+	-	+	-	N.D	N.D	N.D	N.D
Wang X (2014)[48]	M (46)	+	+	-	+	N.D	N.D	N.D	N.D

Ishihara T (2014)[49]	M (60)	+	+	-	-	-	+	-	Partly improved
Nishina T (2018)[50]	M (52)	+	-	-	+	N.D	+	-	Improved

§ All reviewed articles are listed in Supplementary References; Supplemental Digital Content 2

These are included in the main items of the Graus ALE criteria[51]

Abbreviations: CSF: cerebrospinal fluid, EEG: electroencephalogram, F: female, LE: limbic encephalitis, M: male, N.D: not described, +: yes, -: no
